# Trauma Immediately Preceding REM-Behavior Disorder: A Valuable Prognostic Marker?

**DOI:** 10.3389/fneur.2021.710584

**Published:** 2021-11-24

**Authors:** Stevie R. Williams, Nelly Henzler, Pavla Peřinová, Ian A. Morrison, Jason G. Ellis, Renata L. Riha

**Affiliations:** ^1^Sleep Research Unit, Centre for Clinical Brain Sciences, University of Edinburgh, Edinburgh, United Kingdom; ^2^The Department of Neurology and Clinical Sciences, Charles University, Prague, Czechia; ^3^Department of Neurology, University of Dundee, Ninewells Hospital, Dundee, United Kingdom; ^4^Northumbria Centre for Sleep Research, Northumbria University, Newcastle, United Kingdom

**Keywords:** RBD, trauma, REM-behavior disorder, dream enactment behaviors, DEB

## Abstract

**Background:** The definition of rapid eye movement (REM) sleep behavior disorder (RBD) has varied over the years. Rapid eye movement sleep behavior disorder can be considered isolated or idiopathic or can occur in the context of other disorders, including trauma-associated sleep disorder (TSD) and overlap parasomnia. However, whether trauma in RBD carries any prognostic specificity is currently unknown.

**Study Objectives:** To test the hypothesis that RBD secondary to trauma is less likely to result in the development of neurodegeneration compared to idiopathic RBD (iRBD) without trauma in the general population.

**Methods:** A retrospective cohort study of 122 consecutive RBD patients (103 males) at two tertiary sleep clinics in Europe between 2005 and 2020 was studied. Patients were diagnosed as having iRBD by video polysomnography (vPSG) and had a semi-structured interview at presentation, including specifically eliciting any history of trauma. Patients with secondary RBD to recognized causes were excluded from the study. Patients with iRBD were categorized into three groups according to reported trauma history: (1) No history of trauma, (2) traumatic experience at least 12 months prior to RBD symptom onset, and (3) traumatic experience within 12 months of RBD symptom onset. Idiopathic RBD duration was defined as the interval between estimated onset of RBD symptoms and last hospital visit or death. Follow-up duration was defined as the interval between iRBD diagnosis and last hospital visit or death.

**Results:** In a follow-up period of up to 18 years, no patient who experienced trauma within 12 months preceding their iRBD diagnosis received a diagnosis of a neurodegenerative disorder (*n* = 35), whereas 38% of patients without trauma within the 12 months of symptom onset developed a neurodegenerative illness. These patients were also significantly more likely to have a family history of α-synucleinopathy or tauopathy.

**Conclusions:** The development of RBD within 12 months of experiencing a traumatic life event, indistinguishable clinically from iRBD, did not lead to phenoconversion to a neurodegenerative disorder even after 18 years (mean follow up 6 years). We suggest that a sub-type of RBD be established and classified as secondary RBD due to trauma. Additionally, we advocate that a thorough psychological and trauma history be undertaken in all patients presenting with dream enactment behaviors (DEB).

## Definitions

- RBD patients with no trauma history (Group 1)
- RBD patients with trauma that occurred >12 months before RBD diagnosis (Group 2)
- RBD patients with trauma that occurred <12 months before RBD diagnosis (Group 3).

## Introduction

Rapid eye movement (REM) sleep behavior disorder (RBD) is a REM parasomnia characterized by the loss of normal muscle atonia during REM sleep, in association with complex motor activity that usually represents dream enactment behaviors (DEB) (1, 2). The prevalence of RBD has been estimated to be between 0.5 and 2% in the general population (3, 4), with a higher incidence in men after the age of 50 years (5). Rapid eye movement sleep behavior disorder is categorized as either idiopathic/isolated, occurring in the absence of other diseases, or secondary. In secondary RBD, there are other underlying causes present, such as brain lesions, neurological diseases, or provoking antidepressant medications (2). Idiopathic, or isolated, RBD (iRBD) is increasingly but not uniformly considered an α-synucleinopathy (6, 7). However, not all iRBD patients develop a neurodegenerative disorder (7). Whether this is due to insufficient time for the full clinical characteristics of neurodegenerative disease to develop or a lack of α-synuclein/tau pathology in the brain of these patients or an undisclosed history of trauma is unknown at present.

By way of comparison, a recently described parasomnia, trauma-associated sleep disorder (TSD), has substantial symptomatic and clinical overlap with RBD (8, 9) and is thought to be induced by severe psychological trauma. Only a small number of TSD laboratory cases have been reported in the literature (10). As a result, and due to overlap with RBD, identifying TSD as a distinct parasomnia has remained controversial. Additionally, all TSD cases reported in the literature have been active or ex-military personnel, whereas cases from the general population remain to be described (10).

This study aimed to test the hypothesis that nocturnal dream enactment accompanied by REM sleep without atonia (RSWA) has different prognostic implications in iRBD in the context of trauma, in patients recruited from the general population. Given the overlap between RBD diagnostic criteria and reported TSD symptoms, as well as the association between RBD symptom onset in the context of a traumatic experience in a previously reported small number of iRBD patients (11), we hypothesized that patients from the general population with a history of trauma would be phenotypically dissimilar to patients with iRBD and no recent (<1 year) history of trauma. Twelve months from trauma to DEB was chosen as this was the longest time between trauma exposure and symptom onset reported in the case studies of TSD by Mysliwiec et al. (10). An additional control group of iRBD patients that reported traumatic event exposure prior to 12 months of RBD symptom onset was also included, to ascertain whether timing of trauma was relevant.

## Methods

This was a retrospective, cohort study of consecutive RBD patients referred to tertiary sleep referral centers in the United Kingdom and the Czech Republic between May 2005 and February 2020. Formal ethical approval was not deemed necessary by the local research committee since we utilized only anonymised, secondary data derived as part of standard clinical practice. All research was carried out in accordance with Helsinki criteria (World Medical Association, 2001) (12). In our practice, in Edinburgh, it is standard to take a mental health history and to ask about trauma and adverse adult and childhood experiences as part of the patient interview in any patient presenting with a parasomnia. Furthermore, this work was considered to be a service evaluation whereby the information was taken from routine practice and no new questionnaires/interview questions were introduced to gather this information. The dataset derived in the present manuscript was de-identified prior to it being captured and analyzed and all data are presented in aggregated form. This is standard procedure as laid out in the Caldicott principles.

Patient data were obtained using NHS electronic patient notes accessed through TrakCare (www.intersystems.com/TrakCare), a system used to record clinical data in secondary care (hospital) environments. All data were extracted between January 2019 and March 2020. The patient data obtained were collected between May 2005 and February 2020 by medical health care professionals.

At patient presentation, a semi-structured interview was conducted, documenting clinical characteristics, sleep habits, dream content of any reported dreams and history of trauma. Patients were excluded from this study if neurodegenerative syndromes manifested prior to RBD symptom development or if patients were on antidepressant therapy, or had a diagnosis of narcolepsy, as these patients were considered to have secondary RBD according to the ICSD-3 Diagnostic Criteria defined as follows: *repeated episodes of sleep related vocalizations or complex motor behaviors, documented by polysomnography to occur during REM sleep, with REM sleep demonstrating RSWA, and is not better explained by another sleep disorder, mental disorder, medication, or substance use* (2). Patients were also excluded if they had a diagnosis of overlap parasomnia.

Trauma was defined using the ICD-10 trauma definition: …*a “delayed or protracted response to a stressful event or situation (of either brief or long duration) of an exceptionally threatening or catastrophic nature, which is likely to cause pervasive distress in almost anyone”* (13). For comparative purposes, the DSM-5 criteria (“*actual or threatened death, serious injury, or sexual violence”*) were also used (14). Trauma assessment was undertaken by a single medical professional in a blinded manner. Patients were categorized into three groups: (1) No history of trauma, (2) traumatic experience <12 months prior to RBD symptom onset, (3) traumatic experience >12 months prior to RBD symptom onset. No patient in the iRBD group had been diagnosed with post-traumatic stress disorder previously or on presentation. A family history of an alpha-synucleinopathy, dementia/tauopathy, or other neurodegenerative condition was also noted.

Rapid eye movement sleep behavior disorder duration was defined as the interval between self-reported/estimated onset of RBD symptoms and last follow-up visit or death. Follow-up duration was defined as the interval between RBD diagnosis and last follow-up visit or death. All RBD patients met full International Classification for Sleep Disorders-3 criteria (2). Patients with RSWA considered secondary to other etiologies, e.g., medication, were not included in this study.

Patients underwent overnight video polysomnography (vPSG) within 1–6 months of presentation. Video-PSGs were scored by registered sleep physiologists using AASM guidelines (15).

Patients were included in the study if any of the following instances of REM without atonia were present:

- Excessive sustained Chin EMG activity of 2x Stage R atonia was observed for at least 50% of an epoch of REM.
- 50% of 3-s mini epochs contained bursts of transient Chin or Leg EMG activity (0.1–5 s) at least four times greater than Stage R atonia.

The RSWA index (percentage of Stage R with atonia) was not calculated, as this optional reporting statistic was added into AASM Scoring Manual at version 2.6 (2020), after data collection for this study had taken place. The AASM Scoring Manual Version 2.6 has an extra rule regarding EMG tone in REM—Any Chin EMG >2 times Stage R atonia was observed (between 5 and 15 s), and uses 2x not 4x for transient muscle activity. The methods used in this study reflect the fact that version 2.4 was used for analysis of data.

Statistical analysis was undertaken using IBM SPSS Statistics for Windows (Version 24.0. Armonk, NY: IBM Corp.). The Kolmogorov-Smirnov test was used to assess for normality. For discrete variables, the Chi-square test was used. For continuous variables, one-way ANOVA and the Kruskal-Wallis tests were used. Results are reported as number and percentage or mean ± standard deviation (sd). Kaplan-Meier analysis was used to assess risk of developing α-synucleinopathies, and the Mantel Cox test was used to compare the survival curves between groups. All tests were two-tailed where appropriate for subgroup comparisons, and significance was set at *p* ≤ 0.05. As this was a finite population and as there are no previous studies differentiating these populations, power calculations were not considered necessary.

## Results

The RBD cohort overall comprised 103 men (84.4%) and 19 women (15.6%), with a mean age of estimated RBD onset of 53.7 ± 13.9 years (range: 19–76 years). The mean age at RBD diagnosis was 59.1 ± 12.1 years (range: 25–89 years) and the mean duration of RBD prior to presentation was 5.7 ± 4.4 years (range: 0.5–13 years). Thirty-seven patients (30.3%) reported trauma <12 months preceding RBD symptom onset, 14 patients (11.5%) reported trauma >12 months preceding RBD symptom onset, with all meeting the ICD-10 classification of trauma and 53% meeting the stricter standard DSM-V criteria for trauma (Table 1). The remaining 71 patients (58.2%) did not report any traumatic life events. Clinical characteristics across the groups are shown in Table 2. All four patients who had a medical history of sleepwalking had onset in childhood and their NREM parasomnia did not recur in adulthood.

**Table 1:** Type of traumatic event and time at which it was experienced in relation to iRBD onset.

**Trauma type (ICD-10 criteria)**	**Trauma greater than 12 months prior to symptom onset (*n* = 14)**	**Trauma within 12 months of RBD symptom onset (*n* = 37)**
Physical or sexual abuse	21% (*n* = 3)	16% (*n* = 6)
Witness of the death of a loved one^*^	14% (*n* = 2)	16% (*n* = 6)
Operation^*^	7% (*n* = 1)	11% (*n* = 4)
War related	7% (*n* = 1)	3% (*n* = 1)
Road traffic accident	0% (*n* = 0)	3% (*n* = 1)
Near drowning	29% (*n* = 4)	0% (*n* = 0)
Assaulted	0% (*n* = 0)	3% (*n* = 1)
Severe personal, family or work-related stress^*^	14% (*n* = 2)	26% (*n* = 9)
Not disclosed	7% (*n* = 1)	26% (*n* = 9)

*Traumatic event types experienced by patients*.

* *Events not classified in DSM V as trauma*.

**Table 2:** Differences in clinical characteristics across groups diagnosed with iRBD.

**Clinical characteristics**	**No trauma** **(*n* = 71)**	**Trauma greater than 12 months prior to RBD symptom onset (*n* = 14)**	**Trauma within 12 months of RBD symptom onset (*n* = 37)**	***P value***
Male:Female ratio (*n* = 122)	62:9	11:3	30:7	0.57
Estimated age at RBD onset (years) (*n* = 56)	56.21 ± 10.85	49.87 ± 7.18	54.61 ± 14.19	0.42
Age at RBD diagnosis (years) (*n* = 114)	60.92 ± 10.44	54.50 ± 9.36	57.53 ± 15.28	0.13
Duration of RBD (years) (*n* = 115)	5.62 ± 4.81	7.43 ± 4.16	5.09 ± 3.68	0.34
AHI (<5 normal, <15 Mild OSA), <30 Moderate OSA, ≥30 Severe OSA (*n* = 103)	20.99 ± 18.15	28.35 ± 37.21	12.42 ± 10.64	0.08
BMI (*n* = 106)	29.54 ± 5.51	31.17 ± 8.59	28.49 ± 5.71	0.40
Smoker (*n* = 67)	14:25 (56%)	3:6 (50%)	5:14 (36%)	0.77
Alcohol use disorder (DSM V) (*n* = 116)	4 (6%)	0 (0%)	1 (3%)	0.58
Constipation (*n* = 84)	5 (14%)	1 (7%)	1 (3%)	0.47
Self-report of anosmia or changes in sense of smell (*n* = 84)	6 (14%)	3 (21%)	4 (15%)	0.87
Family history of alpha-synucleinopathies (*n* = 109)	8 (14%)	3 (27%)	0 (0%)	0.05
Subsequent diagnosis of neurodegenerative disorder	26 (37%)	6 (43%)	0 (0%)	0.01

*Differences in clinical characteristics between groups (Group 1, n = 71; Group 2, n = 14; Group 3, n = 37). Results are reported as mean ± standard deviation or number and percentage*.

**Figure 1:**
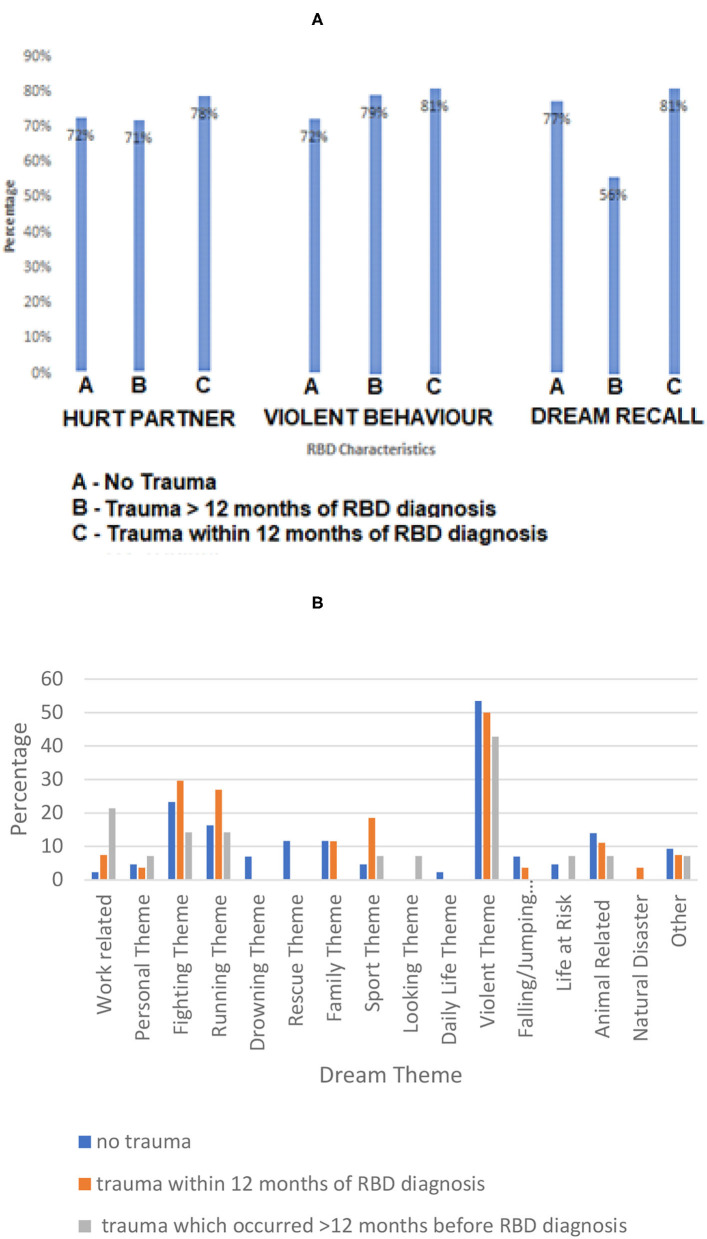
RBD characteristics **(A)** and dream themes **(B)** across groups. **(A)** RBD characteristics for RBD patients with no trauma history (Group 1); trauma which occurred within 12 months of RBD diagnosis (Group 3); trauma which occurred >12 months before RBD diagnosis (Group 2). Percentage of patients is relative to trauma group for **(A,B)**. No significant difference between groups was found in the reported dream recall, incidence of violent nocturnal behavior or incidence in which a bed partner was hurt (*p* > 0.05, data not shown). **(B)** Differences in self-reported dream themes for RBD patients between groups (no trauma history; trauma which occurred prior to RBD diagnosis.). No significant difference was found in the percentage of dream themes between groups (*p* > 0.05, data not shown). Error bars are excluded as bars represent strict counts.

Incidents of violent nocturnal behaviors (e.g., kicking, punching, biting, and scratching) or occasions during which the bed partner was hurt did not differ significantly across the three groups (see Figure 1A). Likewise, there was no significant differences across groups in terms of dream themes (see Figure 1B; *p* > 0.05, data not shown). Both trauma groups reported dreams related to past experiences.

There were no significant differences in REM sleep latency or any additional PSG variables across trauma groups (Table 3). Measurement parameters of autonomic activity (time point and length of measurement) have not been specified by Mysliwiec et al. (8) and so could not be replicated in our study.

**Table 3:** Differences in PSG variables across iRBD groups.

**PSG variables**	**No trauma** **(*n* = 71)**	**Trauma greater than 12 months prior to RBD symptom onset (*n* = 14)**	**Trauma within 12 months of RBD symptom onset (*n* = 37)**	***P*-value**
REM sleep latency (min)	137 ± 78.72	117 ± 65.92	106 ± 68.92	0.45
Sleep latency (min)	45 ± 35.60	41 ± 32.29	44 ± 74.03	0.16
WASO (min)	120 ± 54.15	105 ± 42.27	102 ± 55.36	0.28
REM sleep (%)	15 ± 7.49	18 ± 9.50	19 ± 9.20	0.10
Stage N1 (%)	7 ± 10.29	5 ± 7.39	6 ± 8.28	0.67
Stage N2 (%)	60 ± 17.38	66 ± 16.33	56 ± 16.79	0.19
Stage N3 (%)	10 ± 11.76	5 ± 5.56	13 ± 12.20	0.05
Sleep efficiency (%)	64 ± 14.58	66 ± 16.43	68 ± 18.34	0.26

*Differences in PSG variables across groups (no trauma history, n = 53; trauma which occurred >12 months prior to RBD diagnosis, n = 13; trauma which occurred within 12 months prior to RBD diagnosis, n = 30). Results are reported as mean ± standard deviation or number. PSG variables did not differ significantly across groups (p > 0.05)*.

Self-report of anosmia or recent reduction in sense of smell was 15% across all groups at presentation, with no statistically significant difference across groups (Table 2).

**Figure 2:**
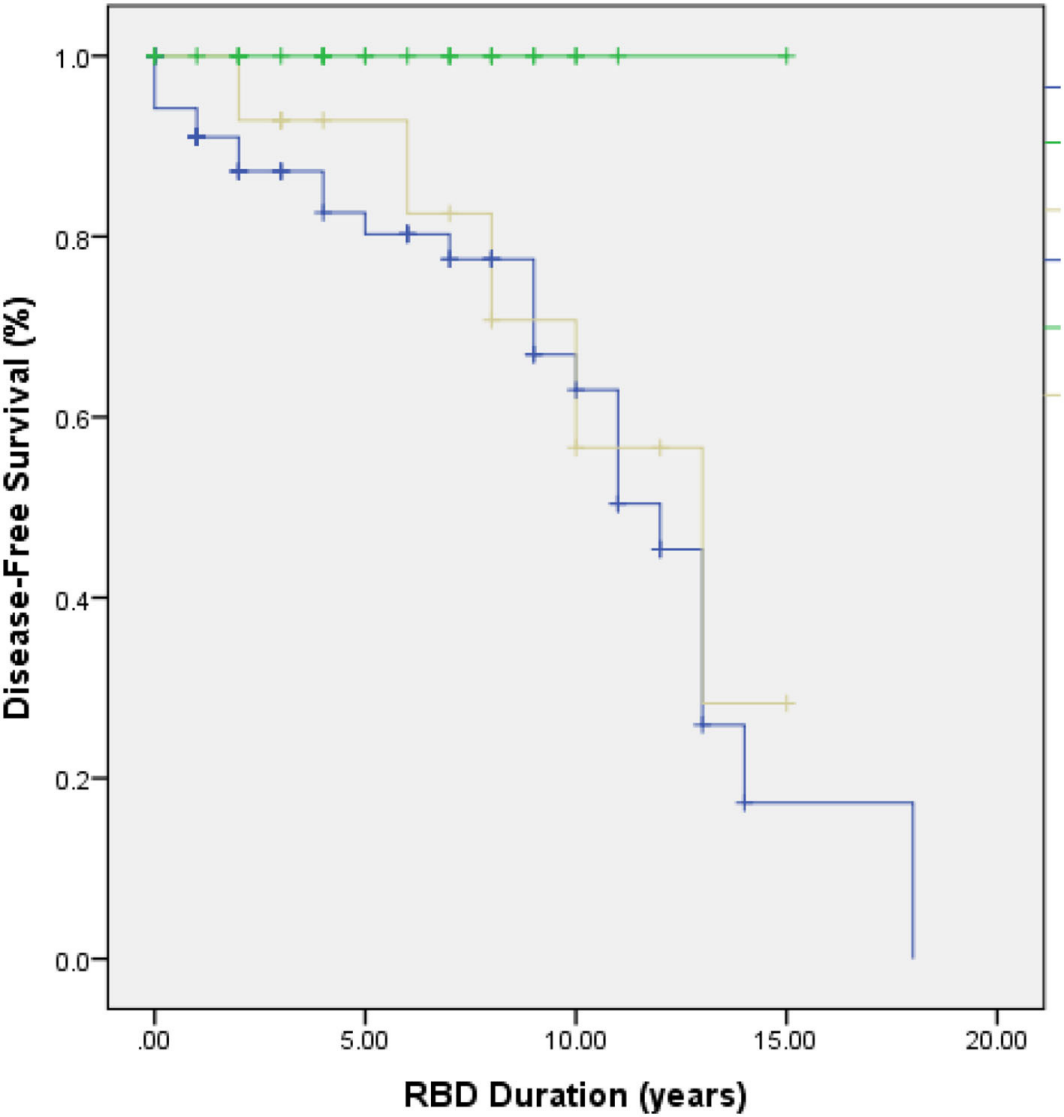
Kaplan-Meier plot of neurodegenerative disease-free survival in Group 1 (blue line), Group 2 (yellow line), and Group 3 (green line). Disease-free survival in RBD patients differed significantly across groups (*p* = 0.01).

In our cohort, 32 patients (26.2%) received a diagnosis, in each case verified by a specialist neurologist, of a neurodegenerative disease after a mean follow-up duration of 870 ± 1,109 days (range: 0.5–18 years) from symptom onset. Disorders that manifested during follow-up (which were not present at RBD diagnosis) were: Parkinson's disease (*n* = 20), Lewy Body dementia (*n* = 7), Progressive supranuclear palsy (*n* = 1), Alzheimer's disease (*n* = 2), and Motor neurone disease with frontotemporal dementia (*n* = 2). Of these patients, 26 (81.3%) had no trauma history (PD *n* = 15, DLB *n* = 5, PSP *n* = 1, AD *n* = 2, MND *n* = 2), and the remaining 6 (18.7%) patients experienced trauma longer than 12 months prior to RBD symptom onset (PD *n* = 6). No patients in Group 3 received a neurodegenerative disease diagnosis during follow-up (see Figure 2), whereas 36.6% of patients in Group 1 and 42.9% of patients in Group 2 went on to develop a neurodegenerative disorder. A significant difference was found in neurodegenerative disease development across groups (*p* = 0.01).

No patients in Group 3 had a family history of neurodegenerative disorders, whereas eight patients (14%) in Group 1 and three patients (27%) in Group 2 had a family history of α-synucleinopathies (*p* = 0.05); see Table 2.

## Discussion

This is the first study to our knowledge to report in detail on trauma associated with “iRBD” onset. Because all patients were phenotypically indistinguishable at presentation and fulfilled criteria for iRBD clinically, we argue that a history of trauma when clearly established as occurring within 12 months of dream-enactment behavior, mandates that it be classified as secondary RBD. Our results demonstrate a significant difference in the disease-free survival rate of patients who had experienced trauma within 12 months of RBD onset as compared to those who had never experienced trauma, or who had experienced historical trauma, prior to RBD onset. At present, it is debatable whether the cohort presented in this study represents “non-military” TSD, as none had a formal diagnosis of PTSD despite reporting often very severe and impactful experiences.

Longitudinal studies have consistently demonstrated that many RBD patients go on to develop an α-synucleinopathy or (less commonly) tauopathy (16). Postuma et al. reported a conversion rate to a neurodegenerative syndrome of 6.3% per year after diagnosis (17) and found 73.5% patients converted after 12-year follow-up. Similar results were found in several other longitudinal studies of RBD cohorts summarized in a recent systematic review and meta-analysis (18). Currently, no reports of phenoconversion to neurodegenerative disease in TSD patients exist. In our cohort, no patients in Group 3 and 38% of patients in Groups 1 and 2 were diagnosed with a neurodegenerative disorder. As the follow-up period for most patients in our cohort was under 10 years, this result may be inconclusive; it is possible that more patients may go on to develop a neurodegenerative disease, if the estimates are correct (17, 19). Nevertheless, our observation has important implications for the understanding of the pathological mechanisms of RSWA and iRBD in the context of trauma and for phenotyping patients appropriately in clinic as well as discussing prognosis.

Our iRBD patient cohort was comprised predominantly of men, consistent with previous findings (17, 18). The reason for this sex difference is unclear. A plausible explanation may be referral bias, as clinical expression of RBD in males has been reported to be more violent and vigorous than in females (11), making males more prone to seek medical attention. Until this study, no female patients had been reported with trauma associated RBD (10).

Rapid eye movement sleep behavior disorder patients younger than 40 years often have secondary forms of RBD (1, 2). Trauma-associated sleep disorder patients reported in the literature are also considerably younger (8), which may be attributable to the demographics of the military. Thirteen percent of patients with recent trauma in our cohort were under 40 years of age, as compared to 3% with historical or no trauma.

In this study, no differences in sleep latency, sleep efficiency, or sleep architecture across groups were found, although both the recent trauma group and historical trauma group had decreased REM latency compared to the group without trauma. These findings, particularly in relation to REM, are interesting considering increased REM sleep latency has been found in fear conditioned rats and mice (20). Fear conditioned mice also have decreased REM epochs (21) and REM length (22). Alternatively, in the absence of aversive stimuli, animals display increased percentages of REM sleep, and duration during the sleep period (21). Fear conditioned animals have been proposed as models for trauma (20), suggesting that trauma affects REM sleep architecture, which may explain the decreased REM latency reported in TSD and the two groups with trauma in our study (8). Unfortunately, human studies are less conclusive: although REM latency may be affected by trauma, in our study, REM latency was decreased. While some PSG studies of traumatized individuals with and without PTSD report REM sleep abnormalities (23), others do not (24).

Invoking the studies on TSD when considering trauma, symptoms of autonomic hyperarousal are included them as part of the diagnostic criteria for TSD (10). Yet only one incident of hyperarousal is documented in TSD patients in the literature and measurement parameters are not specified (8). For this reason, autonomic activity measurements could not be determined in our study using any valid/validated criteria, and our patients should therefore be considered as having RBD secondary to trauma. During normal REM sleep, there is marked suppression of sympathetic activity (25) and in previous reports, RBD patients have been shown to lack autonomic reactivity such as tachypnea and tachycardia despite vigorous limb movements (1).

Our data concurs with findings from previous RBD studies in that dream content mainly involves defense against people or animals and escape from life-threatening situations (11, 26). We found no significant differences in dream themes across groups. Interestingly, patients with and without a trauma history reported dreams that replayed prior life experiences. This has not been reported previously for RBD patients (6, 11), however is consistent with reports in TSD (10). Although in both cases dreams are unpleasant, RBD dreams tend to have similar themes unrelated to past experiences, whereas TSD dreams involved accounts of previous, personal events (10). This type of personal trauma-associated nightmare has been extensively reported in patients with post-traumatic stress disorder (27), indicating that these nightmares are possibly a consequence of trauma.

Reports also suggest that TSD involves more extensive and vigorous movements than RBD (10). We found no differences in violent nocturnal behaviors or incidents in which bed partners were hurt between iRBD patients that experienced trauma and patients with no trauma history. Furthermore, none of the patients displayed disruptive nocturnal behavior during non-REM sleep. One potential explanation may be because patients with concomitant disorders of arousal are likely to attract a diagnosis of overlap disorder and were not included in the cohort analyzed.

We found lower percentages of anosmia or a reduction in sense of smell at diagnosis, and of constipation than reported by Postuma et al. (17), which may be related to our reliance on self-report without objective assessment. Constipation prevalence was 8% across groups, also significantly lower than in the 2019 multicenter study (17).

Functional neuroimaging in humans has found changes in volumetric and cerebral blood flow patterns in wakefulness and sleep, following traumatic event exposure (28). Structures affected include several brainstem nuclei implicated in REM sleep generation and the amygdala (28) which is critical for the processing of fear consolidation, emotional distress, and fear extinction, and is hyperactive in humans following a traumatic experience (29). Liu et al. found that fear conditioning in mice resulted in increased activity in the raphe nucleus and locus coeruleus via the amygdala, with increased twitching during REM sleep (30). This disruption of REM sleep is thought to occur via maintained inhibition of cholinergic activity (21). Animal studies have also shown that projections from the amygdala may generate vocalizations via activation of the central gray matter (31). Thus, the possibility that the amygdala may contribute to RSWA may be a direction for future research. However, only a small number of laboratory studies on the effects of trauma on sleep disturbances exist, and current animal models do not effectively mirror human symptoms (21).

Some limitations of this study must be noted. Healthy controls with and without trauma are necessary to ensure there is no independent association between trauma and neurodegeneration. To the best of our knowledge, no studies have previously tested such a link. Secondly, a major limitation was that trauma was used as a biomarker. A temporal correlation between traumatic experience and RBD symptom onset does not indicate causation in all cases i.e., in some patients, RBD onset may have occurred regardless of traumatic experience. Thus, using an alternative measure, such as autonomic hyperarousal which occurs exclusively in TSD and not RBD would have been beneficial. This was not possible for this study as set thresholds for autonomic hyperarousal measures do not appear to exist. Thirdly, trauma reports relied on patient self-report and individuals may feel reluctant to recount their traumatic event due to resistance to re-experiencing emotion associated with the event (32). Therefore, the actual number of traumatized RBD patients may be higher than self-reported in this study. Not all participants were formally assessed by a psychiatrist as part of standard clinical care but all participants at our clinics are made aware that psychological, mood, and traumatic life experiences may have a bearing on their sleep and sleep quality so are more likely to disclose important information in this regard. Lastly, it is important to note that psychological trauma is very common in the community. In Scotland alone, a population-wide survey published in 2019 showed that 15% of people had suffered significant adverse childhood experiences, with 7% reporting sexual abuse in childhood (33).

In our cohort, 32 patients (26%) developed a neurodegenerative disorder during the follow-up period (range: 0.5–18 years from diagnosis), with a mean RBD duration of 5.7 ± 4.46 years. However, RBD can precede neurodegeneration by more than 20 years, and phenoconversion has been reported to be as high as 80% (19). More patients may be expected to phenoconvert to a neurodegenerative disorder in the future, and longitudinal follow-up of the patients is ongoing. Finally, this was a retrospective cohort study with some data subject to individual recall bias, although bed partners assisted in completing the sleep history wherever possible. Currently, we are prospectively documenting phenoconversion in our growing cohort of patients. Additionally, the validation of sensitive and specific biomarkers will allow us to ascertain better our clinical observations in the very near future (34).

Finally, scoring criteria that are forever being revised present another limitation to studies in this area, making comparisons to older studies more difficult. It is important to point out that the AASM Manual for Scoring Sleep for Associated Events added two new rules regarding RSWA in the latest version (version 2.6), after data collection and sleep scoring of this cohort was completed. The new additions change both the amplitude of EMG required to score phasic events, and importantly insert an optional reporting measure of RSWA %, which is a potential biomarker in predicting neurodegeneration.

In conclusion, we found that the development of RBD clinically with RSWA on polysomnography within 12 months of experiencing a traumatic life event has not led to phenoconversion to a neurodegenerative disorder to date. Patients presenting with the emergence of DEB in the context of recent trauma were also unlikely to have a family history of α-synucleinopathies or tauopathies. We suggest that a sub-type of RBD classified as secondary RBD due to trauma be established. Lastly, this study also highlights the importance of a family history of neurodegeneration as a prognostic marker of earlier decline in iRBD, as has been previously noted (35, 36). Long-term follow-up of the cohort continues.

## Data Availability Statement

The raw data supporting the conclusions of this article will be made available by the authors, without undue reservation.

## Ethics Statement

Ethical review and approval was not required for the study on human participants in accordance with the local legislation and institutional requirements. Written informed consent for participation was not required for this study in accordance with the national legislation and the institutional requirements.

## Author Contributions

RR: concept, statistics, writing, and editing. SW and NH: data collection, statistics, and writing. IM: concept and editing. JE: editing. All authors contributed to the article and approved the submitted version.

## Conflict of Interest

The authors declare that the research was conducted in the absence of any commercial or financial relationships that could be construed as a potential conflict of interest.

## Publisher's Note

All claims expressed in this article are solely those of the authors and do not necessarily represent those of their affiliated organizations, or those of the publisher, the editors and the reviewers. Any product that may be evaluated in this article, or claim that may be made by its manufacturer, is not guaranteed or endorsed by the publisher.

## Abbreviations

AD: Alzheimer's disease
AHI: apnoea hypopnea index
BMI: body mass index
DLB: dementia with Lewy bodies
EMG: electromyography
iRBD: idiopathic RBD
LPT: lateral pontine tegmentum
LDTN: laterodorsal tegmental nucleus
LC: locus coeruleus
MCRF: medullary magnocellular reticular formation
MSA: multiple systems atrophy
PD: Parkinson's disease
PPT: pedunculopontine
PPT: peduncupontine
PSG: polysomnography
PC: precoeruleus
PTSD: post-traumatic stress disorder
RBD: rapid eye movement sleep behavior disorder
RSWA: REM sleep without atonia
RN: red nucleus
SCN: spinal cord neuron
SC: subcoeruleus
SN: substantia nigra
TSD: trauma-associated sleep disorder
vlPAG: ventrolateral periaqueductal gray
vPSG: video polysomnography
WASO: wake time after sleep onset.
